# Effects of Hair Follicle Stem Cells on Partial-Thickness Burn Wound Healing and Tensile Strength

**DOI:** 10.29252/ibj.24.2.99

**Published:** 2019-10-26

**Authors:** Azar Babakhani, Malihe Nobakht, Hamidreza Pazoki Torodi, Mostafa Dahmardehei, Paria Hashemi, Javad Mohajer Ansari, Parisa Ramhormozi, Abazar Yari, Fatemeh Heidari

**Affiliations:** 1 Department of Anatomy, Faculty of Medicine, Iran University of Medical Sciences, Tehran, Iran;; 2 Physiology Research Center, Faculty of Medicine, Iran University of Medical Sciences, Tehran, Iran;; 3 Zahedan Medical University, Zahedan, Iran;; 4 Department of Anatomy, Faculty of Medicine, Alborz University of Medical Sciences, Karaj, Iran;; 5 Department of Anatomy, Faculty of Medicine, Qum University of Medical Sciences, Qum, Iran

**Keywords:** Burn wound healing, Epithelization, Tensile strength, Wound healing

## Abstract

**Background:**

The recent improvements in wound healing have led to new strategies in regenerative medicine. Burn wound healing is an important issue in skin regeneration and has multiple indications for stem cell therapy. HFSCs are a highly promising source of stem cells for healing use, as these cells are accessible, active and pluripotent adult stem cells.

**Methods:**

HFSCs of the rat whisker were isolated, cultured, and labeled with DiI. Flow cytometry method was used to detect special markers of HFSCs. Deep partial-thickness burn wound was created, and labeled HFSCs were injected around the wound bed. Wound closure was recorded via digital photographs. The inflicted rats were sacrificed at 3, 7, or 14 days post burn and used for subsequent histological and tensiometry analysis.

**Results:**

Our results indicated that HFSCs were positive for Nestin and CD34 markers, but negative for Kr15. Morphological and histological photographs revealed that wound closure rate was accelerated in stem cell-treated group compared with other groups. In addition, faster re-epithelialization and collagen deposition were observed. The immunohistochemical analysis suggested that CD31 expression and vascular density enhanced in the stem cell-treated group. Further, tissue tensile strength increased in HFSCs-treated rats in comparison to the control group.

**Conclusion:**

The present study demonstrates that HFSCs could accelerate burn wound healing as well as tensile strength in rats.

## INTRODUCTION

Burn wound repair is a serious challenge in the field of cutaneous wound healing^[^^1^^-^^4^^]^. Partial-thickness burn is one of the most frequent burnings that occurs following contact with hot liquids and steam, hot solids (contact burns) or flames (flame burns), and other electrical or chemical sources^[^^2^^,^^3^^]^. These injuries affect the epidermis and structures beneath the epidermis such as blood vessels, hair follicles, and nerves^[^^3^^]^. Partial-thickness burns are commonly associated with pain, scar, and mood disturbance, and even with death and disability. In severe burns, exposure to high temperature leads to tissue damage. Following the initial tissue damage, the healing process occurs that involves four stages: formation of granulation tissue (granulation), collagenation (collagen deposition), re-epithelialization, and contraction^[^^3^^,^^4^^]^. However, healing of the wound in severe burns remains a major problem with severe complications and high cost of therapy^[^^5^^]^. Therefore, this type of burns require efficient and effective treatments^[^^4^^]^. 

Replacement of damaged skin and incorporation of skin appendages such as hair follicles, sebaceous glands, sweat glands, and other accessory organs in addition to blood vessels and nerves are several indications for stem cell therapy in severe burns^[^^6^^,^^7^^]^. HFSCs are easily accessible multipotent stem cells that can be used in burn wound healing. These stem cells have proved to be capable of proliferating rapidly and generating a stratified epidermis on human burn wounds. Besides, they have a critical role in epidermal homeostasis, turnover, and maintenance^[^^6^^,^^8^^]^. It has also been demonstrated that HFSCs can contribute to wound closure and repair^[^^9^^]^. Subsequently, studies have suggested that HFSCs yield epidermal cells, endothelial cells, hair follicle cells, keratinocytes, and a broad range of cell types with specialized functions in the body^[^^6^^,^^10^^]^. HFSCs have a long lifespan and high degree of physiological plasticity *in vitro*. In addition, in contrast to other types of adult stem cell, bulge HFSCs do not raise ethical concerns and are free of immune rejection (to prevent graft-versus-host disease), making them candidate for regenerative medicine and cell replacement therapies^[^^11^^]^. Accordingly, we decided to examine, for the first time, the potential effect of transplantation of HFSCs on burn wound healing (for which morphological and histological assay was used) and tensile strength *in vitro*.

## MATERIALS AND METHODS

**Rat HFSCs isolation and culture**

In this experimental study, adult male Wistar rats (180-200 g, n = 10) were sacrificed for isolation and culture of HFSCs using a previous protocol with a minor modification^[^^9^^,^^12^^]^. Briefly, the whisker of the upper lip of each rat was removed and placed in collagenase I/dispase II solution (Sigma, USA) for 10 min. Then the vibrissa follicles were lifted out and rinsed from around the connective tissue. The bulges were removed from the capsule, and two transverse cuts were made above and below the follicle for dissecting the bulge region. Next, the small bulge pieces were cultured in collagen type I-coated tissue culture flasks (25 cm^2^). The flasks containing the culture medium consisted of a 3:1 DMEM/F12 (containing 10% of FBS, 100 U/ml of penicillin, and 100 µg/ml of streptomycin, 0.1 U/ml of insulin, and 0.5 mg/ml of hydrocortisone) were kept in 95% air, 5% CO_2_ at 37 °C. Five to six days after cell attachment to the collagen in tissue culture flasks, bulge fragments were lifted out, and cells were incubated in the same medium culture. All the surgical procedures and cultivation were performed under sterile conditions. The animal procedures used in this study were approved by the Ethics Committee of Iran University of Medical Sciences (Tehran), and all the processes were performed in accordance with the university’s indices.

**Flow cytometric analysis**

Flow cytometric analysis was used to determine the percentage of stem cell expressing the special marker. For this purpose, the cells were detached with 0.25% Trypsin-EDTA. Next, the cell plaque was suspended in a fixation medium and incubated for primary antibodies against the CD34 (1:75), Nestin (1:200), and Kr15 (1:75), antigens (all from Sigma) at 37 °C for 1 h. Then the cells were incubated with a secondary antibody conjugated by FITC (1:1400, Sigma) in the dark at 37 °C for 1 h. The incubated cells without primary antibody were considered as the negative control.

**DiI-labeled HFSCs**

1,1’-dioctadecyl-3,3,3’,3’-tetramethyl indocarbocy-anine perchlorate (DiI, Sigma) was used for HFSCs labeling before cell transplantation. The cells were detached with Trypsin-EDTA 0.25% and centrifuged for 10 min. Then 1 × 10^6^ cells were exposed to 2 μg of DiI/ml medium at 37 °C for 30 min and then were washed twice by PBS (for removing excess dye) and centrifugation steps were followed. The labeled stem cells were injected intradermally around the burn wound at four injection sites (at 12, 3, 6, and 9 o’clock positions) using an insulin syringe with a 31-gauge needle (Avapezeshk Company, Iran). The rats were then sacrificed, tissue samples were collected, and a fluorescent microscope (Olympus AX70, Japan) was used to check the result.

**Animal care and creation of burn wounds**

**Fig. 1:**
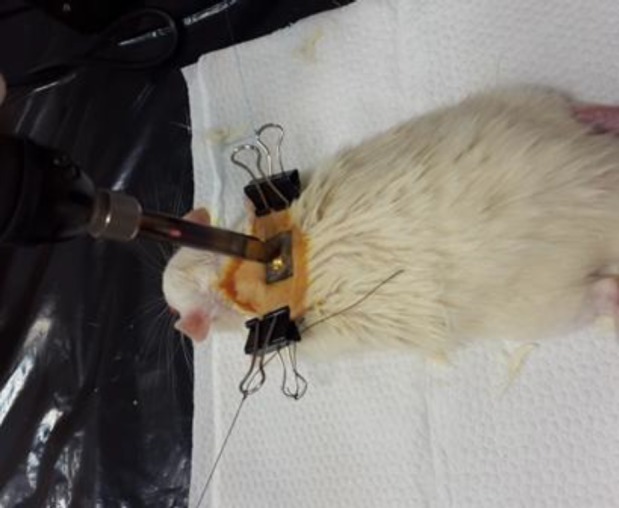
Creation of burn wound model using two clips upward and outward away from the underlying viscera. The heated plate was replaced perpendicular to the skin, resting on its own weight

A total of 45 adult Wistar male rats (200-250 g; Pasteur Institute of Iran, Tehran) were housed in the animal house of Iran University of Medical Sciences for one week before wounding. All animal care and experimentations were in accordance with the guidelines and authorization of the Institutional Review Board and the Institutional Ethical Committee of Iran University of Medical Sciences. The rats were kept in a 12 h light/dark cycle and received water and food. They were randomized into three equal experimental groups (n = 15 in each group): (1) cell or treatment group; (2) control or lesion group without any treatment; (3) sham or PBS group. The male Wistar rats were anesthetized with ketamine (50-60 mg/kg) and xylazine (6.5-7.5 mg/kg), where the dorsum of the rats was shaved and disinfected. According to the thermal burn method described in previous studies^[^^2^^,^^13^^,^^14^^]^, burn induction was performed by direct conduction burn model using a 65 W soldering iron with the tip and square tip 15 × 15 mm (iron plate) at optimized 150 °C temperature. The heated instrument was positioned vertically under its own weight (85 g) and held in contact with the skin for 10 seconds (Fig. 1), in order to create a deep partial-thickness burn^[2]^. The electronic temperature was controlled by a soldering station (RX-711AS, Goot, Japan). Using two clips, the portion of skin to be burnt was pulled upward and outward, away from the underlying viscera.

**Wound analysis**

The progressive changes in the wound area were measured. Digital photographs of burn wounds were taken on the day of burn creation as well as on days 3, 7, and 14 post burning using a Canon Power Shot SX100 camera (Canon, USA). ImageJ software was then used for analysis. The evaluation was performed by investigators blind to the treatment protocol and groups. The percentage of wound closure was calculated as follows: %wound closure = (A0-Ai)/A0 ×100^[^^9^^,^^15^^]^, where A0 is the area of day 0 and Ai is the area of the indicated day.

**Histological assay**

Tissue samples with a surrounding rim of normal skin were collected on days 0, 3, 7, and 14 for histological studies. Paraffin sections of 5-µm thickness were stained by H&E and Masson’s trichrome and further examined under light microscopy.

**Immunohistochemical assay**

Fluorescent immunostaining was performed for examining the expression of CD31 in the burned sample from each group on days 7 and 14. The aim was to detect whether angiogenesis had occurred in the wound bed (dermis). The paraffin-embedded tissue sections were incubated with rabbit monoclonal anti-CD31 (1:100, Abcam, USA) antibody at 4 °C overnight. Then the sections were incubated with the goat anti-rabbit secondary antibody (1:1000, Abcam) at 37 °C for 1 h and developed with 3,30-diamino-benzidine tetrahydrochloride solution and counterstained with hematoxylin. The lumen of blood vessels appeared brown.

**Tensiometry**

On day 14 post burn, the tensile strength determination was performed. The skin was cut into strips of 12 cm in length, 5 cm in width, and 3.5 cm in thickness, where the wounded part was in the center. Then the strips were fixed into the tensiometer holder (Zwick 72.5, German) and elongated from zero length at the constant speed of 10 mm/min until they were ruptured. Tissue stress/strain was then evaluated.

**Statistical analysis**

The results were presented as the mean ± standard deviation. Data analyses were performed by one-way ANOVA, followed by Tukey's post-hoc test. Statistical analysis was conducted using Prism 6 for windows. For all tests, a *p* value less than 0.05 was regarded as statistically significant.

## RESULTS

**Isolation and cultivation of HFSCs**

In the present study, bulge HFSCs from dissected rats were successfully isolated and cultured with a minor modification. The adherent cultured HFSCs began to extend from the isolated bulge (Fig. 2A) on 3-4^th^ days of cultivation and then formed dome-like colonies around the bulge segments (Fig. 2A and 2B). Gradually, with rapid proliferation, after 7-9 days, the cells initiated to migrate out of the colonies, with a homogeneous population of cells, enclosing the bottom of the flask after nine days (Fig. 2C and 2D). The cells reached confluency in 2-4 days and then were subcultured to other collagen-coated flasks in the same medium.

**Fig. 2:**
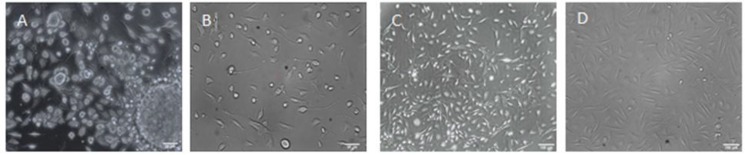
The primary culture of bulge HFSCs from rat hair follicles. (A) HFSCs 3-4 days after the primary culture; (B and C) migration and proliferation of HFSCs after the colony formation; (D) HFSCs culture after nine days (scale bar A and B = 20 µm; C and D = 100 µm)

**Flow cytometry**

To confirm that the extracted bulge cells of the rat vibrissa follicle were primitive stem cells, flow cytometry was utilized. The results indicated that the bulge cells were CD34 and Nestin-positive but Kr15-negative. The expressions of the cell surface markers of CD34, Nestin, and Kr15 were 70%, 75%, and 12.5%, respectively (Fig. 3).

**Wound healing assay**

We decided to evaluate the HFSCs effect on deep partial-thickness burn wounds heaing. The results obtained from morphological examinations suggested that the rat wounds implanted with HFSCs exhibited an enhanced wound closure (Fig. 4A), and healing of the burn area on days 7 and 14 significantly improved (*p* < 0.001), compared to the rats treated with PBS alone and untreated control wounds (Fig. 4B). The results also revealed that the burn closure process was significantly faster in HFSCs group with a mean wound closure of 72.61 ± 1.44% compared with the control group with a mean wound closure of 46.36 ± 1.40 on day 14. However, there was no significant difference between the PBS and control groups on day 14 with a mean wound closure of 52.68 ± 2.43 and 46.36 ± 1.40, respectively.

**Histological and immunohistochemical analysis**

Histological analysis was used to evaluate tissue regeneration. The results indicated that the epidermal layer was completely formed and fully covered the wound site in the HFSCs-treated group 14 days post implantation. However, in the control and PBS-treated controls, the re-epithelialization was not fully completed (Fig. 5A). Also, the results demonstrated that the length of the newly regenerated epidermal layer and its thickness was significantly higher for the stem cell-treated group, probably due to the presence of HFSCs at their site of action (Fig. 5B). In addition, the thickness of granulation tissue and newly regenerated dermis in stem cell-treated group was higher than that of the PBS and control groups on day 7 post implantation. Meanwhile, wound maturity was observed in the central and marginal parts of stem cell-treated wounds. According to the results of evaluation of hair regeneration (Fig. 6B), on day 14, we clearly observed hair follicles wrapped by sebaceous glands in the stem cell-treated group. However, in the PBS-treated and control groups, some messy and not-yet mature follicles began to appear. Newly formed blood vessels are necessary for tissue regeneration. On day seven, newly formed blood vessels could be visualized across all the three groups (Fig. 6A), while in the control group, blood vessel density in both the stem cell-treated and PBS groups was far higher (Fig. 6A). Surprisingly, mature vessels were clearly observed in the stem cell group on day 14.

**Fig. 2:**
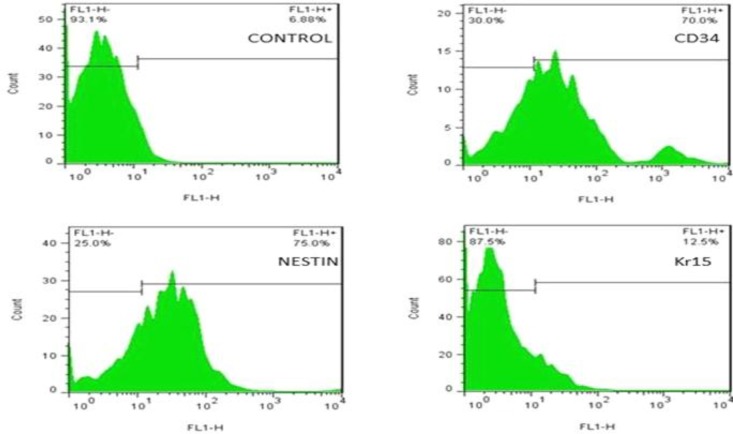
Flow cytometry assay from the surface adhesion molecules on HFSCs with nestin, CD34, and Kr15 antibodies before differentiation. Flow cytometry results indicate the percentage of CD34-positive, nestin-positive, and Kr15-negative cells. Incubated cells with only secondary antibody have been considered as the negative control

**Fig. 4:**
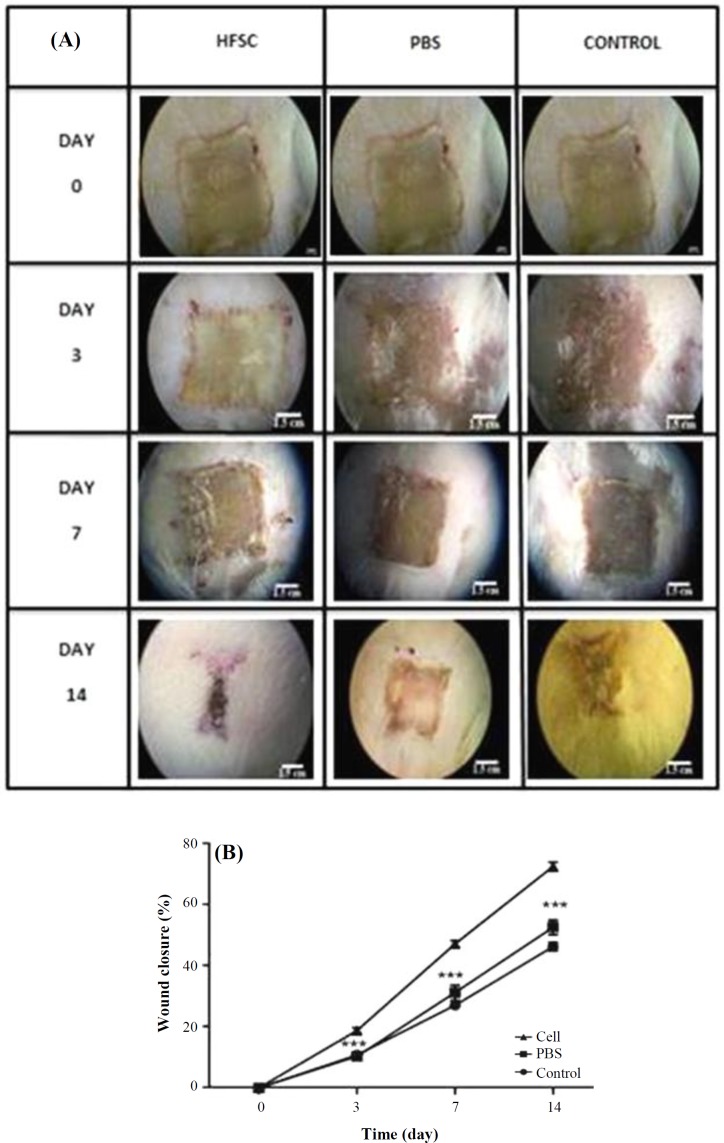
The effects of HFSCs on burn wound closure. (A) Photographs of the wounds on days 3, 7, and 14 post burn, respectively; (B) wound healing analysis of HFSCs, PBS, and control groups on different days. Analysis of variance versus control (^***^p < 0.001)

**Fig. 5:**
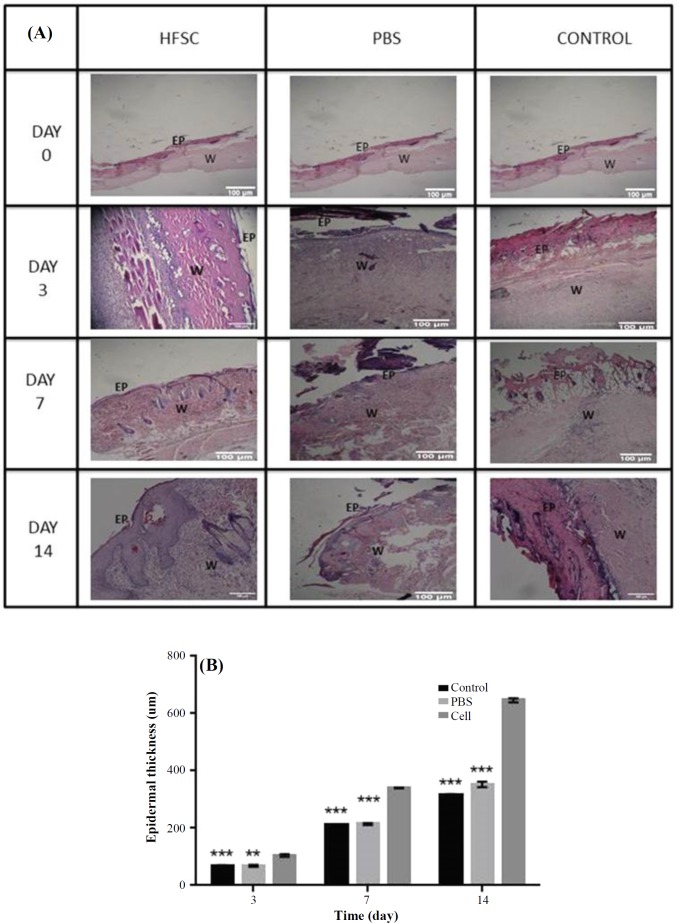
Histological analysis of burn wounds in rat skin. (A) The processed tissue was assessed microscopically after H & E staining in HFSCs, PBS, and control groups; (B) epidermal thickness (n = 6, on days 3, 7, and 14; analysis of variance, mean ± SD; ^***^p < 0.001 for all, except cell vs. PBS on day 3: ^**^p < 0.01); EP, epidermis; W, wound bed

Analysis of Masson’s trichrome-stained section demonstrated the formation and deposition of collagen fibers during the first seven days post burning (Fig. 7A). However analysis of collagen fiber density suggested that collagen formation and deposition was faster in the stem cell-treated group compared to all others (Fig. 7B). Collagen fiber density as well as collagen accumulation and deposition was higher in the HFSCs-treated group, with more regular arrangements compared with the control and PBS groups on day 14. Furthermore, collagen deposition in the wounds treated with HFSCs was relatively comparable to that found in normal skin, showing that dermal regeneration was enhanced using HFSCs. However, collagen formation was delayed, and no specific organization of collagen fiber was identified in the PBS and control groups at the same time.

The ability of HFSCs for angiogenesis (neovascularization) was evaluated by immuno-histochemistry analysis for CD31 on days 7 and 14 post burning. As observed in Figure 8, newly CD31-expressed vessels could be clearly identified in stem cell-treated group on day seven. Over time, a large number of CD31-expressing mature vessels immediately were observed clearly below the epidermis (Fig. 8). These results further indicated that HFSCs can promote the formation of blood vessels, concurrent with the onset of the matrix remodeling phase.

**Fig. 6:**
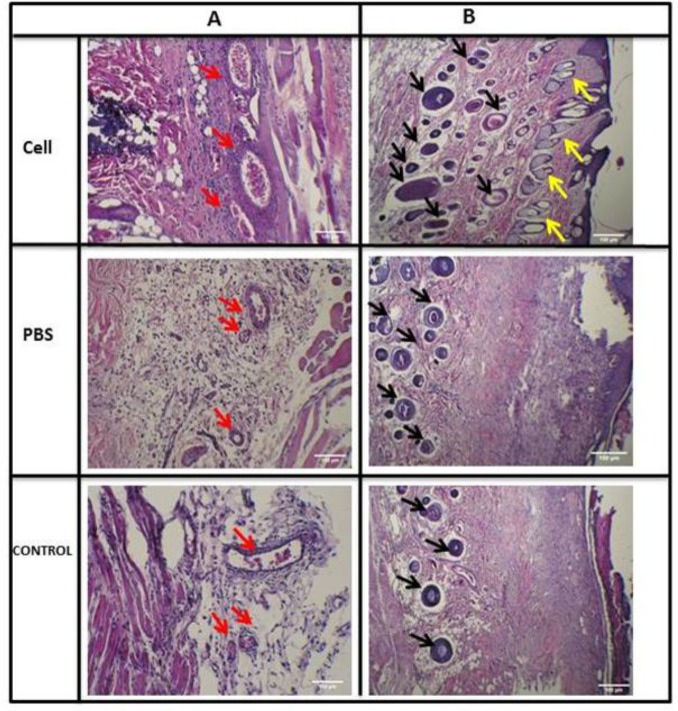
(A) The angiogenic effects of HFSCs in burn wound observed in H & E sections in HFSCs, PBS, and control groups (red arrows: blood vessels); (B) effects of HFSCs on hair follicles and sebaceous glands regeneration found in H& E sections in HFSCs, PBS, and control groups (black arrows: hair follicles and yellow arrows: sebaceous glands).

**Fig. 7:**
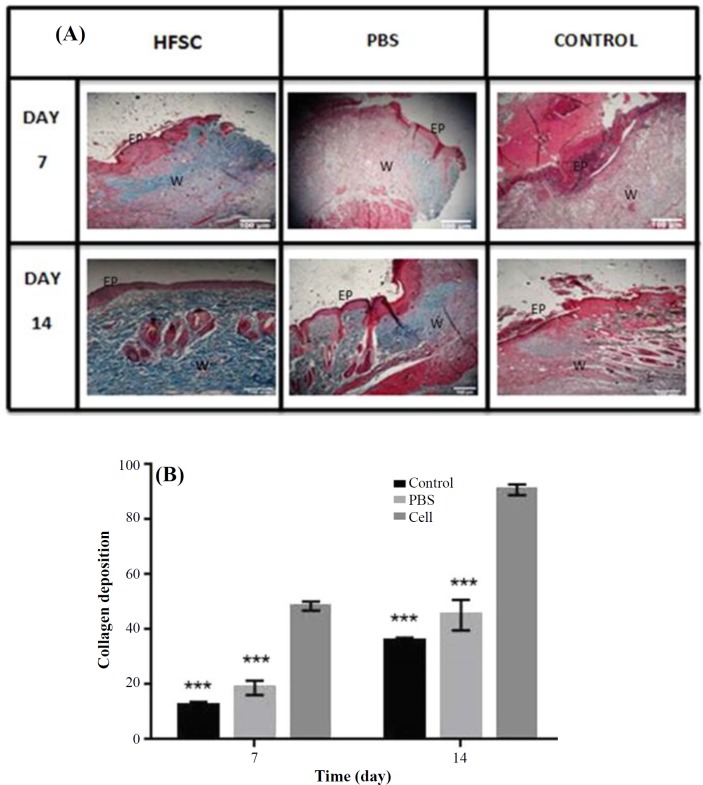
Histological analysis of burn wound healing in rat skin with Masson’s trichorome stain. (A) The processed tissue was assessed microscopically after Masson’s trichorome in HFSCs, PBS, and control groups at 7 and 14 days post burn; (B) Histological analysis of collagen deposition on days 7 and 14 after wound induction (n = 6; analysis of variance, mean ± SD; ^***^p < 0.001); EP, epidermis; W, wound bed

**Tracking the transplanted DiI-labeled HFSCs**

In this study, burned wounds treated with labeled HFSCs were analyzed on days 3, 7, and 14 post transplantation. The results indicated that the transplanted HFSCs survived throughout the experimental time (day 14) and migrated to the epidermis (day 3) and then to the dermis (on days 7 and 14), as shown in Fig. 9A-E.

**Tensiometry**

The tissue stress (maximum force tensile leading to tissue rupture) and tissue strain (tissue length under maximum pressure) in stem cell-treated group on day 14 post burning significantly enhanced in comparison with other groups (*p* < 0.001; Fig. 10).

## DISCUSSION

Burn is a severe trauma in normal anatomical structure of skin with loss of its natural function. Despite major advances in burn treatment, burn wound healing has remained a serious problem, for which innovative treatment is necessary. Stem cell therapy is a promising new treatment for burns, which involves applying stem cells to repair damaged tissue. Stem cell therapy following burn injury may offer an alternative treatment strategy not only for wound healing but also for treating systemic effects of burn injury^[^^6^^,^^16^^]^. 

**Fig. 8:**
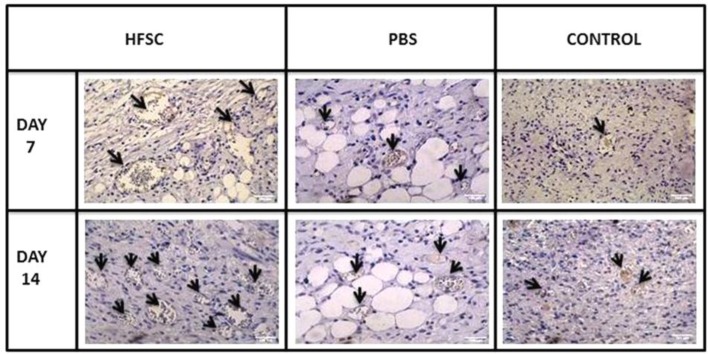
The angiogenesis activity of the HFSCs detected by immunohistochemical staining for CD-31 expression (brown) vessels (arrows) in wound tissue in HFSCs, PBS, and control groups on days 7 and 14 post burn

Application of stem cells is a new area in tissue regeneration field. Regenerative medicine aims both to enhance re-epithelialization after burn injury and to renovate functional skin via an efficient stem cell therapy^[^^17^^]^. HFSCs as one of the most popular adult stem cells found in the hair follicle bulge are currently explored for regenerative medicine. The advantages of these stem cells include easy availability, immunomodulatory and non-oncogenic effects, no ethical concerns and multilineage differentiation^[^^6^^,^^16^^]^. Bulge stem cells react rapidly with epidermal wounding through producing short-lived Transit-Amplifying cells responsible for acute wound healing^[^^18^^]^. Also, epidermal and dermal stem cells can contribute to skin repair and be stimulated into endothelial and neural lineages^[^^6^^,^^19^^,^^20^^]^. Therefore, people affected by burns and other wounds would benefit from HFSCs therapy products. 

In this study, HFSCs were isolated and cultured successfully and specified by flow cytometry, through the presence of stem cell markers CD34 and nestin, but not the keratinocyte marker Kr15 (Fig. 3). Moreover, a single dorsal skin burn (deep partial-thickness) was inflicted in experimental rats through transferring energy (heat) using direct conduction (iron/skin) by a controlled temperature (150 °C) for 10 seconds. Previous studies have reported several methods for creating burn wounds in rats. Applying heated instruments is one of the most widely used procedures. In this regard, an ideal experimental protocol, which would achieve burn wound, is consistent in size and depth in the temperature (60-200 °C) and exposure time (8-15 seconds)^[^^1^^,^^2^^,^^21^^-^^23^^]^. Further, because these models prevented skin contraction, it resulted in uniform wound closure^[^^22^^]^. In this study, we found that the subdermal transplantation of HFSCs in the wound area caused accelerated burn wound closure; therefore, wound closure rate significantly enhanced in stem cell-treated group compared with other groups. These results are in agreement with Heidari *et al*.'s^[^^9^^]^ study suggesting that HFSCs accelerated excisional-wound closure. Cutaneous wound healing involves well-coordinated integration of cell migration and proliferation, along with collagen formation, re-epithelialization, hair follicle regeneration, angiogenesis, and remodeling^[^^24^^]^. The results of the HFSCs treatment on the re-epithelialization process revealed that the length of the newly regenerated epidermal layer and epidermal thickness were significantly elevated, possibly due to the presence of the HFSCs in their site of action. Further, in the HFSCs group, the epithelial layer was completely formed and covered the whole burn wound site on day 14. Dense organized dermis with thick granulation tissue and many functional blood vessels and abundant mature hair follicles wrapped by sebaceous glands were observed in all assayed days in the stem cell-treated group compared with the other groups. Regeneration of hair follicles and sebaceous glands plays an important role in wound healing and functionality of renewed skin^[^^4^^,^^16^^]^. It has been proven that well-effective angiogenesis and sufficient blood supply at the site of burn injury are two main factors affecting the burn healing process, and neova-scularization is critical to preserve the regenerated granulation tissue. There is also a relationship between angiogenesis and expression of CD31^[^^15^^]^, a transmembrane glycoprotein expressed on the surface of endothelial cells^[^^14^^]^. In this study, to evaluate the effects of HFSCs transplantation in angiogenesis, immunohistochemistry analysis was performed for the CD31 antibody. The results indicated a high-grade neovascularization activity in the specimens from HFSCs-treated group, especially on days 7 and 14 post treatment, compared with the other groups. Nevertheless, some stained blood vessels in brown color were observed in the PBS group. Consistent with these finding, recent studies have confirmed that creation and development of new vessels during the early wound healing process enhanced^[^^7^^,^^9^^,^^14^^]^, possibly due to the presence of stem cells. These newly vessels could decrease the inflammation phase and provide the nutrients and oxygen for cell proliferation and tissue regeneration^[^^7^^,^^9^^,^^14^^]^. Angiogenesis activity of HFSCs and improved overall wound healing process have also been reported in Heidari *et al.*^[^^9^^]^ and Xu *et al.*^[^^14^^]^ studies. 

**Fig. 9:**
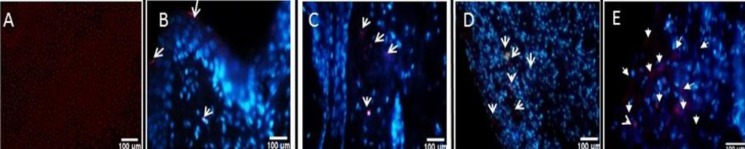
Imaging of HFSCs migration in burn wounds. (A) DiI-labeled HFSCs; (B) DiI-labeled HFSCs three days post burn and treatment in the epidermis; (C) labeled HFSCs seven days post burn in the dermis; (D and E) labeled HFSCs 14 days post burn in the center of the burn wound. Arrows represent labeled HFSCs

**Fig 10:**
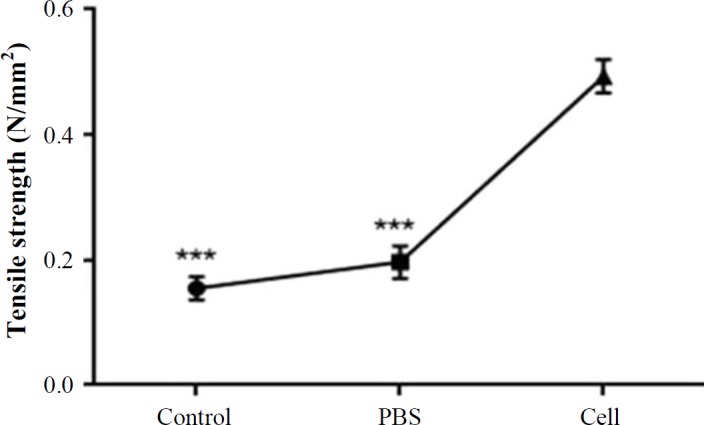
Tensile strength analysis of burn wound samples of HFSCs, PBS, and control group on day 14 post burn (n = 6; analysis of variance; mean ± SD, ^***^p < 0.001).

Collagen synthesis and deposition are important for cutaneous wound healing as well as for development of wound strength. The results of Masson’s trichrome staining revealed that HFSCs have positive effects on the deposition and correct orientation of collagen fibers (but not scar formation) in the burned area in the stem cell-treated group on day 14 post burn. We also found that tissue tensile strength, the breakdown strength per unit of cross-sectional area describing the healing rate of the wound increased in rats receiving HFSCs in comparison to the control group. Tensile strength is one of the most important factors used in the wound healing studies^[^^5^^,^^25^^,^^26^^]^. It is a valuable measure that reflects the subdermal organization of the collagen fibers in a newly granulated tissue^[^^5^^]^. Biomechanical strength examination confirmed the results of Masson’s trichrome staining. We also found that direct injection of HFSCs (subdermal injection) can be considered an alternative to transplantation. HFSCs labeled with fluorescent dye showed survival and existence in the epidermal, dermal, and the center of burn wound, respectively, indicative of no sign of immunorejection over 14 days post transplantation. These finding are consistent with previous results of Heidari *et al*.^[^^9^^]^ study. The results of the present study suggested that treatment with HFSCs significantly enhanced the healing of deep-partial thickness burn wound and tensile strength in rats. These findings indicate that autologous HFSCs could be applied for burn wound repair in inflicted patients.
